# Factors to consider during anesthesia in patients undergoing preemptive kidney transplantation: a propensity-score matched analysis

**DOI:** 10.1186/s12871-023-02208-8

**Published:** 2023-08-05

**Authors:** Jeayoun Kim, Kyo Won Lee, Keoungah Kim, Hyeryung Kang, Jaehun Yang, Jae Berm Park, Gaabsoo Kim

**Affiliations:** 1 Department of Anesthesiology and Pain Medicine, Samsung Medical Center, Sungkyunkwan University School of Medicine, 81 Irwon-Ro, Gangnam-Gu, Seoul, 06351 Korea; 2 Department of Transplantation Surgery, Samsung Medical Center, Sungkyunkwan University School of Medicine, Seoul, Korea; 3 Department of Anesthesiology and Pain Medicine, School of Dentistry, Dankook University, Cheonan, Korea; 4 Department of Surgery, Gil Medical Center, Gachon University College of Medicine, Incheon, Korea

**Keywords:** End-stage renal disease, Intraoperative hypotension, Living donor kidney transplantation, Metabolic acidosis, Preemptive kidney transplantation

## Abstract

**Background:**

International guidelines have recommended preemptive kidney transplantation (KT) as the preferred approach, advocating for transplantation before the initiation of dialysis. This approach is advantageous for graft and patient survival by avoiding dialysis-related complications. However, recipients of preemptive KT may undergo anesthesia without the opportunity to optimize volume status or correct metabolic disturbances associated with end-stage renal disease. In these regard, we aimed to investigate the anesthetic events that occur more frequently during preemptive KT compared to nonpreemptive KT.

**Methods:**

This is a single-center retrospective study. Of the 672 patients who underwent Living donor KT (LDKT), 388 of 519 who underwent nonpreemptive KT were matched with 153 of 153 who underwent preemptive KT using propensity score based on preoperative covariates. The primary outcome was intraoperative hypotension defined as area under the threshold (AUT), with a threshold set at a mean arterial blood pressure below 70 mmHg. The secondary outcomes were intraoperative metabolic acidosis estimated by base excess and serum bicarbonate, electrolyte imbalance, the use of inotropes or vasopressors, intraoperative transfusion, immediate graft function evaluated by the nadir creatinine, and re-operation due to bleeding.

**Results:**

After propensity score matching, we analyzed 388 and 153 patients in non-preemptive and preemptive groups. The multivariable analysis revealed the AUT of the preemptive group to be significantly greater than that of the nonpreemptive group (mean ± standard deviation, 29.7 ± 61.5 and 14.5 ± 37.7, respectively, *P* = 0.007). Metabolic acidosis was more severe in the preemptive group compared to the nonpreemptive group. The differences in the nadir creatinine value and times to nadir creatinine were statistically significant, but clinically insignificant.

**Conclusion:**

Intraoperative hypotension and metabolic acidosis occurred more frequently in the preemptive group during LDKT. These findings highlight the need for anesthesiologists to be prepared and vigilant in managing these events during surgery.

## Background

Kidney transplantation (KT) is an established treatment for end-stage renal disease (ESRD) [1]. Recently, international guidelines have recommended preemptive KT, which involves performing the transplantation before initiating dialysis [2, 3]. This approach offers several advantages, including improved graft function and patient survival by avoiding dialysis-related complications such as cardiovascular disease and infection [4–10]. Additionally, preemptive KT has been shown to offer significant benefits in terms of patient welfare [11, 12] and societal cost-saving [13, 14].

Patients undergoing preemptive KT may encounter specific challenges related to ESRD due to the relatively shorter transition period from ESRD to surgery. These challenges encompass imbalances in volume status and metabolic disturbances associated with acid/base and electrolyte levels, such as hyperkalemia, calcium, and phosphate disorders. Additionally, ESRD patients are at an increased risk for perioperative bleeding due to impaired platelet function and platelet-vessel wall interactions caused by the accumulation of uremic toxins [15–18]. Unfortunately, these issues often remain unresolved at the time of surgery.

However, there is a paucity of studies assessing the potential risks during anesthesia in preemptive KT patients. Therefore, we planned to investigate the anesthetic events that occur when patients were undergoing preemptive KT and determine which aspects require further attention. We conducted a retrospective cohort study to test our primary hypothesis that the extent and duration of intraoperative hypotension would be greater in the preemptive group compared to the nonpreemptive group.

## Methods

### Study design

**Fig. 1:**
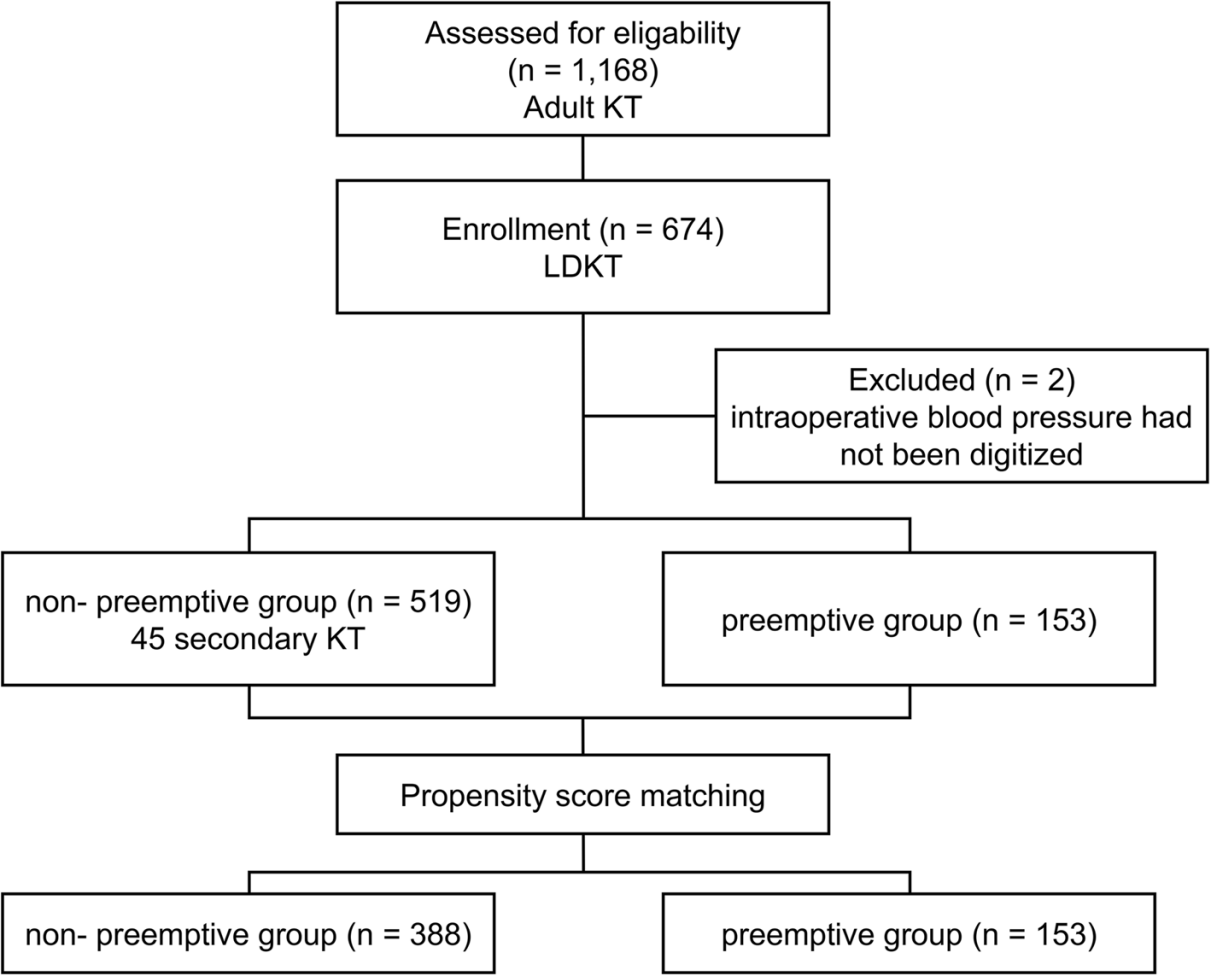
Flow diagram for study enrollment and grouping

The present study was a single-center, retrospective cohort study. The Institutional Review Board of Samsung Medical Center approved this study and waived the requirement for written informed consent (SMC 2018–10-147–001). A total of 1,168 adult recipients, who underwent KT between April 2010 and October 2018 at Samsung Medical Center, was the initially screened cohort. Among these recipients, 494 recipients who underwent deceased donor KT (*n* = 494) and with missing digitized data on intraoperative blood pressure (*n* = 2) were excluded. The remaining 672 recipients who underwent living donor KT (LDKT) were analyzed in the present study, who were divided into the nonpreemptive and preemptive groups (Fig. 1).

### Data collection

Data were obtained from the electronic medical records or a KT database (prospectively collected) and were anonymized/de-identified before analysis. Baseline mean arterial pressure (MAP) was defined as the average of preoperative MAP values measured in the ward noninvasively until the patient was moved to the operating room (maximum of 10 recordings). Intraoperative MAP was recorded every five minutes automatically. We chose the invasive blood pressure values if both invasive and noninvasive blood pressure measurements were available; however, noninvasive blood pressure values were used if the invasive blood pressure could not be relied on due to artifacts. When MAP was not recorded, the MAP was calculated using the systolic and diastolic arterial pressure (SAP and DAP, respectively) according to the following formula: MAP = DAP + 1/3 × (SAP − DAP). Preoperative echocardiographic parameters were collected and used to evaluate the presence of uremic cardiomyopathy which is characterized by ventricular dysfunction and hypertrophy [19]. Left ventricular diastolic dysfunction (LVDD) was assessed according to the clinical comorbidities and echocardiographic findings [20–22]. We classified the patients with ischemic heart disease (IHD), clinical diagnosis of congestive heart failure (CHF), pathologic left ventricular hypertrophy (LVH), or left ventricular ejection fraction < 50% as having LVDD. We defined the cutoff values for LVH as left ventricular mass index > 115 g/m^2^ in men and > 95 g/m^2^ in women [23]. The modality and duration of renal replacement therapy, redo-KT, time from the last dialysis to KT in the nonpreemptive group were also investigated.

Intraoperative arterial level of bicarbonate, base excess, and serum electrolytes (i.e., sodium, potassium, and calcium) were collected from the arterial blood gas analysis which was carried out immediately after induction. Postoperative nadir creatinine (Cr) values (i.e., the lowest Cr level in blood tests conducted according to the postoperative protocol), estimated glomerular filtration rate (eGFR), and Cr clearance (CCl) were collected. Serum and urine Cr level was measured with the kinetic alkaline picrate method (Jaffe Method) and the time to attain nadir Cr was determined via retrospective chart review. The eGFR was calculated using the modification of diet in renal disease equation [24].

### Outcomes

**Fig. 2:**
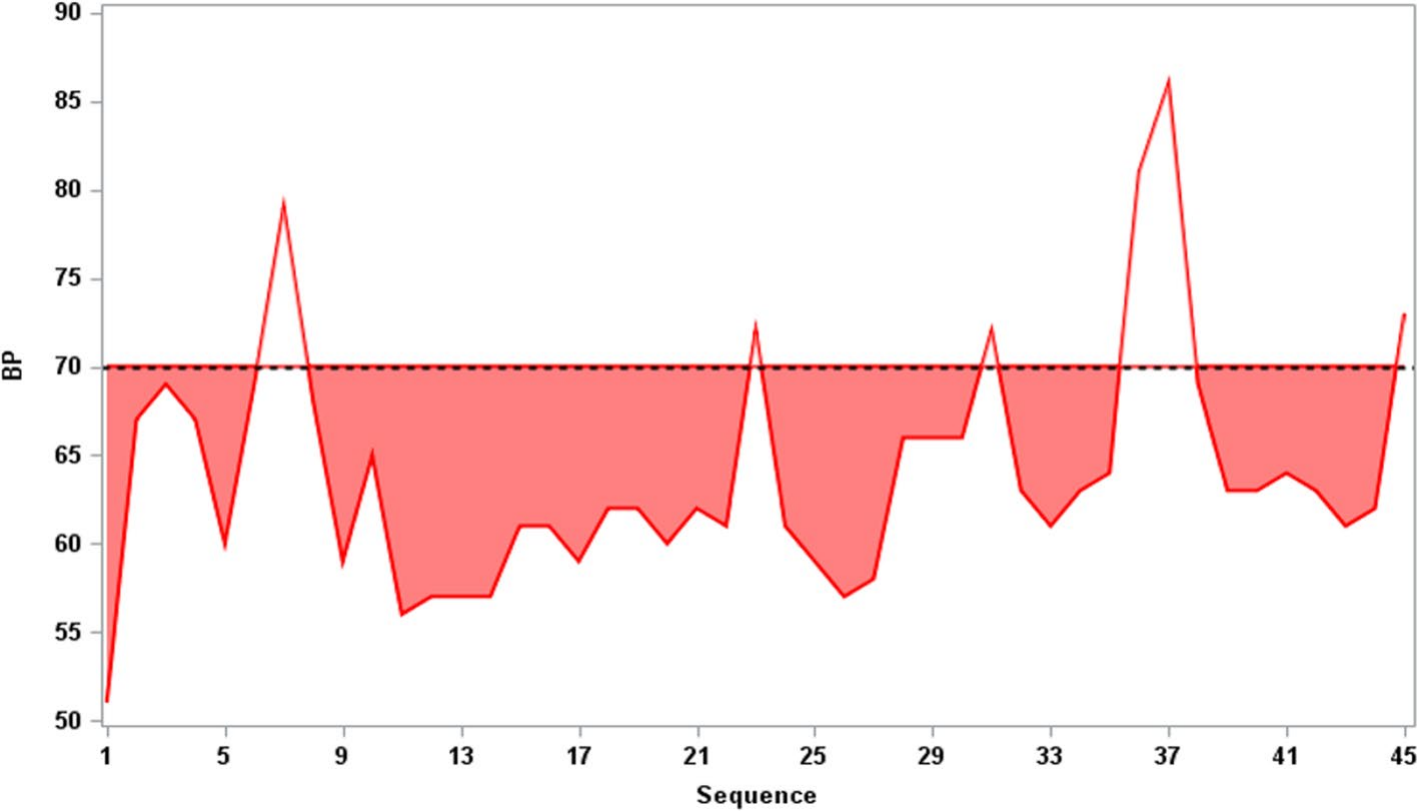
Scheme for calculating area under the threshold

The primary outcome was intraoperative hypotension defined as the sum of the area under the threshold (AUT) on the blood pressure curve. Figure 2 represents AUT calculation. AUT was the area under 70 mmHg in the graph with the X-axis as time and the Y-axis as MAP, reflecting the duration and severity for which an individual's MAP was measured below 70 mmHg. The MAP threshold of 70 was determined according to our institutional protocol and previous meta-analysis on the effect of intraoperative hypotension on end-organ damage [25, 26]. We utilized blood pressure data obtained from the time of surgical incision to graft reperfusion to compute AUT. This approach was implemented to mitigate the influence of various factors that could potentially affect blood pressure levels prior to surgical incision, including endotracheal intubation and positional changes.

Secondary outcomes were intraoperative use of inotropes (such as dopamine or dobutamine), vasopressors (such as norepinephrine or vasopressin), or red blood cell transfusion, total volume of crystalloid fluid and synthetic colloid, immediate graft function as defined by the postoperative nadir Cr value and maximum eGFR, time to achieve nadir Cr, and postoperative transfusion and bleeding which was defined as the need for re-exploration due to bleeding.

### Anesthesia management

Anesthetic management was performed according to a standardized institutional protocol. No premedication was administered. After the patients arrived at the operating room, standard monitors including a non-invasive blood pressure monitor, pulse oximetry, electrocardiogram, and bispectral index monitor (BIS, Aspect Medical Systems, Natick, MA, USA) were applied. After the intubation was performed, invasive blood pressure monitoring was performed via the right radial artery. An antibiotics-coated 7-Fr three-lumen catheter was placed in the right internal jugular vein and central venous pressure was monitored. Anesthesia was maintained using inhalational anesthetics, desflurane. The oesophageal Doppler probe (CardioQ; Deltex Medical, Irving, TX, USA) or FloTrac/Vigileo monitoring system (FloTrac/Vigileo version 3.02, Edwards Lifesciences, Irvine, CA, USA) was applied, which facilitated estimations of preload and afterload. Balanced crystalloid including Hartmann's solution (JW Pharmaceutical, Seoul, Korea) and Plasma solution A (CJ, Seoul, Korea) were primarily used as the maintenance fluid. Fluid infusion and vasoactive/inotropic drugs were adjusted to maintain MAP ≥ 70 mmHg prior to graft reperfusion and ≥ 90 mmHg following graft reperfusion to improve graft perfusion based on the hemodynamic variables in the CardioQ and FloTrac/Vigileo monitoring system. For patients with metabolic acidosis (base excess < -10 mmol/L) and hemodynamic instability despite the use of vasoactive/inotropic drugs, sodium bicarbonate was infused at the clinician’s discretion. Intraoperatively, the target blood hemoglobin for allogeneic red blood cells was 7.0 g/dl.

### Statistical analysis

Statistical analysis was performed using SAS version 9.4 (SAS Institute Inc., Cary, NC, USA) and R version 3.4.3 (R Foundation for Statistical Computing, Vienna, Austria) with the package matchIt. Continuous variables were analyzed using the Wilcoxon rank-sum test or Student’s t-test where appropriate and presented as median (interquartile range) or mean (standard deviation) values, respectively. Categorical variables were analyzed using the chi-squared or Fisher’s exact test where appropriate and presented as numbers (%).

We performed propensity score matching using nearest-neighbor matching without replacement and a matching tolerance of 0.2 to balance baseline characteristics and avoid selection bias, which was triggered by a nonpreemptive:preemptive ratio of 3:1 maximally with the following seven covariates: age, sex, diabetes, hypertension, prescription of antihypertensive medications (Angiotensin converting enzyme inhibitor, angiotensin receptor blocker, beta-blocker, or calcium channel block), and history of CHF and IHD. Successful matching was defined when standardized mean difference for matched variables were < 10% [27]. After matching, 388 of 519 in the nonpreemptive group were matched with 153 of 153 patients in the preemptive group (Fig. 1). the group comparison of patient characteristics and outcomes were conducted using an univariable generalized estimating equation (GEE). The association of potential risk factors with intraoperative hypotension defined by AUT was tested by GEE. Factors with a *p*-value of less than 0.1 in the univariable analysis and group were included in the multivariable analysis. We conducted Spearman’s correlation analysis in the preemptive group to assess the correlation between metabolic acidosis and AUT. A *P*-value of less than 0.05 was considered to be statistically significant.

## Results

The comparison of demographic and clinical parameters between the preemptive group and the nonpreemptive group is shown in Table 1. Prior to propensity score matching, 45 recipient (8.7%) underwent redo-KT. Most patients (473/519, 91.1%) in the nonpreemptive group had received hemodialysis and the median (IQR) duration of renal replacement therapy was 6.1 (2, 34) months, with an average interval of 1.6 days between the last dialysis session and the surgery. The nonpreemptive group exhibited a higher frequency of diabetes and CHF compared to the preemptive group. Conversely, hypertension was more prevalent in the preemptive group. In the matched cohort, the standardized mean differences of all seven matched-covariates were reduced to values below 10%, indicating that an adequate balance was achieved between treatment groups. Echocardiographic parameters suggested that LVH and LVDD were more frequent in the nonpreemptive group compared to the preemptive group. The baseline MAP was not significantly different prior to matching, but after matching, a significant difference was observed between the two groups (99.2 mmHg and 102.0 mmHg, respectively, *P* < 0.0015).

**Table 1:** Patient characteristics

**Characteristics**	**Entire population**				**Propensity-score matched population**			
	**Nonpreemptive**	**Preemptive**	**SMD**	*P* value*	**Nonpreemptive**	**Preemptive**	**SMD**	*P* value**
	**(*n* = 519)**	**(*n* = 153)**			**(*n* = 388)**	**(*n* = 153)**		
Age > 60 years	56 (10.8)	17 (11.1)	0.010	0.911	37 (9.5)	17 (11.1)	0.052	0.649
Body mass index, kg/m^2^	22.6 (20.4, 25.1)	22.8 (20.7, 25.3)	0.038	0.748	22.6 (20.3, 25.1)	22.8 (20.7, 25.3)	0.026	0.917
Female sex, n (%)	211 (40.7)	66 (43.1)	0.050	0.584	159 (41.0)	66 (43.1)	0.044	0.744
Diabetes, n (%)	156 (30.1)	32 (20.9)	0.211	0.027	92 (23.7)	32 (20.9)	0.067	0.406
Hypertension, n (%)	457 (88.1)	140 (91.5)	0.114	0.234	346 (89.2)	140 (91.5)	0.079	0.432
Antihypertensive medication, n (%)	407 (78.4)	138 (90.2)	0.328	0.001	339 (87.4)	138 (90.2)	0.090	0.430
Cerebrovascular disease, n (%)	16 (3.1)	3 (2.0)	0.072	0.587	11 (2.8)	3 (2.0)	0.057	0.534
Congestive heart failure, n (%)	22 (4.2)	0 (0)	0.298	0.010	0 (0.0)	0 (0)	N/A	N/A
Ischemic heart disease, n (%)	29 (5.6)	8 (5.2)	0.016	0.864	18 (4.6)	8 (5.2)	0.027	0.652
Revised cardiac risk index, n (%)			0.232	0.060			0.066	0.930
0	410 (79.0)	134 (87.6)			331 (85.3)	134 (87.3)		
1	92 (17.7)	16 (10.5)			48 (12.4)	16 (10.5)		
2	17 (3.3)	3 (2.0)			9 (2.3)	3 (2.0)		
Echocardiographic parameters^a^								
LVEF, %	62 (57, 66)	63 (59, 67)	0.325	0.021	63 (58, 67)	63 (59, 67)	0.224	0.017
LVMI, g/m^2^	117.2 (99.3, 145.0)	100.9 (89.4, 128.5)	0.455	< 0.001	117.62 (99.9, 147.5)	100.9 (89.4, 128.5)	0.466	< 0.001
LAVI, ml/m^2^	39.3 (31.4, 48.9)	36 (29.8, 42.3)	0.363	0.006	39.75 (32, 48.8)	36 (29.8, 42.3)	0.364	< 0.001
E/A ratio	0.94 (0.71, 1.2)	0.92 (0.74, 1.32)	0.109	0.334	0.95 (0.7, 1.2)	0.92 (0.74, 1.32)	0.085	0.290
Deceleration time, m/sec	222 (193.8, 261.0)	225.6 (205.1, 252.3)	0.040	0.443	220.1 (192.1, 261.0)	225.6 (205.1, 252.3)	0.048	0.567
E', m/sec	0.07 (0.06, 0.08)	0.08 (0.06, 0.1)	0.325	< 0.001	0.07 (0.06, 0.09)	0.08 (0.06, 0.1)	0.239	0.005
E/E' ratio	10 (8, 13)	8.31 (6.6, 10.3)	0.619	< 0.001	9.95 (8.0, 12.4)	8.3 (6.6, 10.3)	0.592	< 0.001
TR velocity (m/sec)	2.4 (2.2, 2.6)	2.24 (2.1, 2.4)	0.529	< 0.001	2.4 (2.2, 2.7)	2.2 (2.1, 2.4)	0.554	< 0.001
Left ventricular hypertrophy, n (%)	300 (64.4)	56 (42.1)		< 0.001	227 (65.0)	56 (42.1)	0.473	< 0.001
Systolic dysfunction, n (%)			0.319	0.055			0.223	0.150
LVEF ≥ 60%	299 (64.2)	96 (71.6)			229 (59.0)	96 (52.7)		
50% ≤ LVEF < 60%	138 (29.6)	37 (27.6)			108 (27.8)	37 (24.2)		
40% ≤ LVEF < 50%	17 (3.6)	1 (0.7)			7 (1.8)	1 (0.7)		
LVEF < 40%	12 (2.6)	0 (0)			5 (1.3)	0 (0)		
Diastolic dysfunction, n (%)^b^			0.484	< 0.001			0.430	< 0.001
Indeterminate	50 (10.7)	5 (3.7)			121 (31.2)	44 (28.8)		
Grade 1	177 (38)	44 (32.6)			38 (9.8)	5 (3.3)		
Grade 2	92 (19.7)	18 (13.3)			72 (18.6)	18 (11.8)		
Grade 3	5 (1.1)	0 (0)			1 (0.3)	0 (0.0)		
Preoperative plasmapheresis for ABOi KT	93 (17.9)	25 (16.3)	0.042		66 (17)	25 (16.3)	0.018	0.858
Modality of renal replacement therapy, n (%)			N/A	N/A			N/A	N/A
Hemodialysis	473 (91.1)	0 (0)			351 (90.5)			
Peritoneal dialysis	46 (8.9)	0 (0)			37 (9.5)			
Duration of renal replacement therapy, months	6 (2, 34)	0 (0, 0)	N/A	N/A	5.03 (1.7, 29.0)		N/A	N/A
Operation time, min	183 (155, 213)	174 (151, 216)	0.093	0.405	184 (155, 213)	174 (151, 216)	0.097	0.422
Baseline Cr, mg/dL	7.7 (5.7, 10.2)	7.5 (6.4, 9.2)	0.089	0.980	8.1 (6, 10)	7.5 (6.4, 9.2)	0.081	0.295

Data are expressed as as mean ± standard deviation (SD), median (interquartile range), or as number (percentage)

*SMD* standardized mean difference, *LV* left ventricular, *EF* ejection fraction, *LAVI* left atrial volume index, *E/A ratio* E/A ratio of peak spectral transmitral flow velocities, *E'* peak early diastolic tissue velocity at the mitral annulus, *TR* tricuspid regurgitation, *ABOi KT* ABO incompatible kidney transplantation, *Cr* creatinine

^*^P values were calculated using Mann–Whitney U test, student t-test, chi-square test, or Fisher's exact test

^**^P values were calculated using generalized estimating equation

^a^72 missing values for LVEF, 73 missing values for LVMI,76 values for LAVI, 97 missing values for E/A ratio, 102 missing values for deceleration time, 86 missing values for E', 97 missing values for E/E' ratio, 249 missing values for TR velocity

^b^71 missing values for diastolic dysfunction

### Anesthetic events

The anesthetic events that occurred during surgery are summarized in Table 2. The generalized estimating equation demonstrated that the mean AUTs was significantly higher in the preemptive group compared to the nonpreemptive group (29.7 ± 61.0 vs. 14.5 ± 37.7, *P* = 0.002). This findings consisted in the multivariable analysis, which adjusted for the effect of baseline MBP and RCRI score (*P* = 0.007). Also, more inotropes and vasopressors were administered in the preemptive group (*P* < 0.001 and = 0.002, respectively).

**Table 2:** Intraoperative outcomes

	**Nonpreemptive**	**Preemptive**	***p* value**
	**(*n* = 388)**	**(*n* = 153)**	
**Primary outcome**			
AUT (< MAP 70 mmHg)^a^	14.5 ± 37.7	29.7 ± 61.0	0.002
**Secondary outcomes**			
Inotropic use, n (%)	54 (13.9)	43 (28.1)	< 0.001
Vasopressor use, n (%)	7 (1.8)	15 (9.8)	< 0.001
Red blood cell transfusion, n (%)	18 (4.6)	17 (11.1)	0.007
Total volume of crystalloid, L	3.1 (2.5, 3.6)	3.1 (2.6, 3.7)	0.127
Total volume of synthetic colloid, L	0.2 (0, 0.5)	0.2 (0, 0.5)	0.675
Initial blood gas analysis^b^			
Base excess, mmol/L	0.6 (-1.9, 2.9)	-7.4 (-10.1, -4.9)	< 0.001
Serum bicarbonate, mmol/L	24.7 (22.5, 26.7)	18.1 (16.1, 19.9)	< 0.001
Serum sodium level, mmol/L	136 (134, 138)	137 (135, 139)	< 0.001
Serum potassium level, mmol/L	4.1 (3.6, 4.6)	4.1 (3.8, 4.6)	0.673
Serum calcium level, mg/dL	1.0 (1.0, 1.1)	1.0 (1.0, 1.1)	0.914
Sodium bicarbonate use, mEq	0 (0, 0)	0 (0, 0)	0.009

^a^AUT (< MAP 70 mmHg), area under the threshold, with a threshold set at a mean arterial blood pressure below 70 mmHg

^b^Blood gas analysis was carried out immediately after induction

**Fig. 3:**
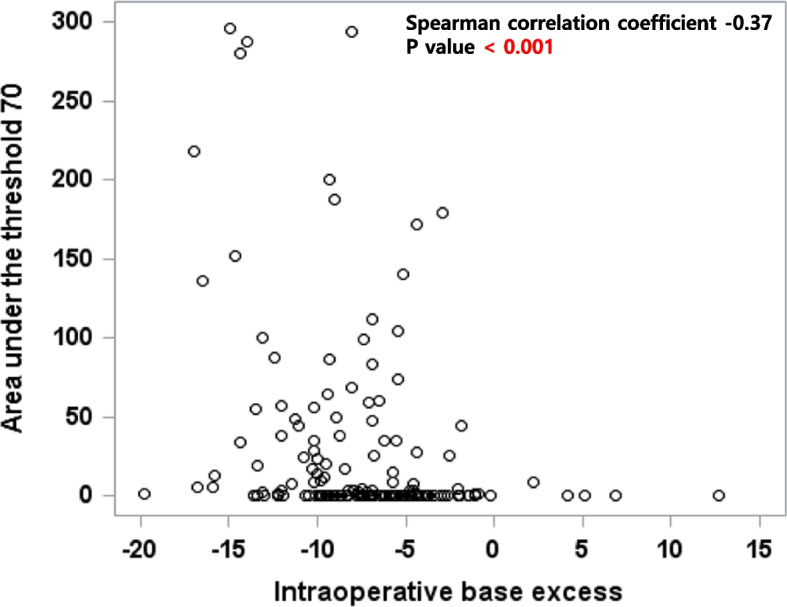
Relationship between intraoperative base excess levels and area under the threshold (AUT) with a threshold set at a mean arterial blood pressure below 70 mmHg

Metabolic acidosis was severe in the preemptive group. Bicarbonate and base excess values in the nonpreemptive were lower and the median (range) dose of intraoperative bicarbonate administered was higher compared to the nonpreemptive group (0 [0, 240] vs. 0 [0, 160], *P* = 0.009). Finally, base excess values demonstrated low negative correlation with AUT in the preemptive group (Fig. 3).

### Immediate postoperative outcomes

The details of the immediate postoperative course following KT is summarized in Table 3.

**Table 3:** Immediate postoperative outcomes

	**Nonpreemptive**	**Preemptive**	***p* value**
		**(*n* = 388)**	**(*n* = 153)**
Nadir Creatinine (mg/dL)	0.96 ± 0.3	0.90 ± 0.34	0.024
Time to nadir Creatinine (day)	5.55 ± 4.1	4.08 ± 2.3	< 0.001
Maximum eGRF (mL/min/1.73m^2^)	89.7 ± 28.4	93.7 ± 27.7	0.099
Maximum Cr clearance	114.7 ± 49.4	130 ± 88.3	0.050
Postoperative transfusion, n (%)^a^	85 (21.9)	45 (29.4)	0.082
Postoperative bleeding, n (%)^b^	5 (1.3)	1 (0.7)	0.528

*Cr* creatinine, *eGFR* estimated glomerular filtration rate

^a^Postoperative transfusion within 3 days

^b^Postoperative bleeding was defined when the recipient required re-exploration because of bleeding

The nadir Cr value in the nonpreemptive group was 0.96 ± 0.3 mg/dL and that in the preemptive group was 0.90 ± 0.34 mg/dL (*P* = 0.0237), while the times to nadir Cr in the nonpreemptive and preemptive groups were 4.08 ± 2.3 and 5.55 ± 4.12 days, respectively (*P* < 0.0001); however, neither of these were clinically impressive, even though the differences were statistically significant. Operation time and postoperative bleeding were also not significantly different between the nonpreemptive and preemptive groups.

## Discussion

This study was designed based on the hypothesis that preemptive KT patients exhibit greater hemodynamic instability during anesthesia and experience worse postoperative graft function compared to nonpreemptive KT patients. In the preemptive group, the extent and duration of intraoperative hypotension were greater than the nonpreemptive group. Immediate postoperative graft function, as indicated by nadir Cr level and time to nadir Cr level demonstrated a clinically insignificant improvement in favor of the preemptive group compared to the nonpreemptive group.

In the present study, the preemptive group exhibited significantly greater duration and severity of intraoperative hypotension as measured by AUT, despite the nonpreemptive group showing a higher frequency of left ventricular hypertrophy, systolic and diastolic dysfunction. The observed significant of these findings remained after adjusting for baseline MAP in multivariable analysis. Our findings contrast with the results reported in a previous study that compared blood pressure at the beginning, in the middle and end of the surgery [28]. We conducted comparative analysis between the two groups, examining the extent and duration of blood pressure below a specific threshold for blood pressure, which was AUT, utilizing blood pressure data measured at 5-min intervals during surgery. Since there is currently no established threshold for intraoperative hypotension, we used a MBP threshold of 70, based on a meta-analysis that identified a threshold associated with acute kidney injury [25]. Considering that not only the severity but also the duration of intraoperative hypotension has been associated with postoperative end-organ damage [25], AUT may serve as more reliable predictors for post-KT outcomes compared to blood pressures measurements taken at specific time points. Therefore, our findings could be more robust and reliable compared to previous studies that reported insignificant difference regarding the intraoperative blood pressure between the two groups [28].

The observed differences in the extent and duration of hypotension, as measured by AUT, between the two groups may be attributed to differences in metabolic acidosis. In the nonpreemptive group, the majority of patients underwent dialysis the day before surgery (478/519, 92.1%), which resulted in a significantly favorable base-excess profile compared to the preemptive group. The preemptive group exhibited significantly lower levels of intraoperative base excess and bicarbonate compared to the nonpreemptive group, and base excess level demonstrated a weak negative correlation with AUT. Metabolic acidosis could lead to reduction in cardiac contractility and catecholamine efficiency, and decreased vascular responsiveness to inotropics and vasopressors through various mechanisms [29–35]. In this regard, more pronounced metabolic acidosis in the preemptive group might have contributed to the occurrence of intraoperative hypotension.

Several studies have reported favorable outcomes in preemptive KT, including higher rates of graft success and lower rates of delayed graft function and acute rejection [4–7, 28, 36, 37]. However, other studies have suggested that the graft outcomes in the preemptive group are comparable to those in the nonpreemptive group [38–41]. In our study, despite the preemptive group experiencing a greater extent and duration of intraoperative hypotension, we observed statistically significant but clinically insignificant differences in the nadir Cr value and time to reach the nadir Cr level, favoring the preemptive group over the nonpreemptive group. Intraoperative hypotension has been associated with an increased risk of postoperative kidney injury, with studies indicating that prolonged exposure (10 min) to MAP below 80 mmHg and shorter exposure to MAP below 70 mmHg are associated with increased risks [25, 26]. Moreover, the extent of intraoperative hypotension has been reported to be an independent risk factor for slower graft function [42]. Given these findings, one might expect a more detrimental impact on graft function and recovery in the preemptive group, where the extent and duration of intraoperative hypotension were greater. However, our study only revealed clinically insignificant differences. This may be attributable to the fact that we analyzed blood pressure data before graft reperfusion and the anesthesiologists made efforts to maintain the blood pressure above a MAP of 100 mmHg after reperfusion to avoid renal hypoperfusion using appropriate fluids and medications until sufficient urine output was achieved [43].

This study has several limitations. Firstly, this study is a retrospective cohort study conducted at a single center. Therefore, the generalizability of our findings to other populations may be limited and causal inference between the preoperative dialysis and intraoperative blood pressure remains uncertain. Future prospective studies or randomized controlled trials are needed to confirm the results obtained in this study. Secondly, although there is standardized anesthesia protocol for LDKT, some anesthesiologists may have opted for their own anesthesia regimen, deviating from the standardized protocol, which may have led to differences in intraoperative blood pressure. Thirdly, while efforts were made to account for all potential variables influencing intraoperative hypotension, there may be unconsidered confounding factors that persist. Fourthly, propensity score-matching resulted in the exclusion of transplant recipients who were expected to be hemodynamically unstable or have poor outcomes. For instances, no recipients in the preemptive group had a history of CHF, leading to exclusion of 22 recipients of the nonpreemptive group with a history of CHF. This suggests a potential risk of skewing the results. Finally, the present study focused on intraoperative and short-term outcomes, and further research should investigate the long-term implications of preemptive KT.

## Conclusion

Intraoperative hypotension and metabolic acidosis occurred more frequently in the preemptive group during LDKT. Our findings highlight the need for anesthesiologists to be prepared and vigilant in managing these events during surgery.

## Data Availability

The datasets used and/or analysed during the current study are available from the corresponding author on reasonable request.

## Abbreviations

AUT: Area under the threshold
CHF: Congestive heart failure
Cr: Creatinine
CCl: Creatinine clearance
DAP: Diastolic arterial pressure
eGFR: Estimated glomerular filtration rate
ESRD: End-stage renal disease
IHD: Ischemic heart disease
KT: Kidney transplantation
LDKT: Living-donor KT
LVDD: Left ventricular diastolic dysfunction
LVH: Left ventricular hypertrophy
MAP: Mean arterial pressure
SAP: Systolic arterial pressure
